# The Five Eras of Chiropractic & the future of chiropractic as seen through the eyes of a participant observer

**DOI:** 10.1186/2045-709X-20-1

**Published:** 2012-01-19

**Authors:** J Keith Simpson

**Affiliations:** 1 School of Chiropractic & Sports Science Murdoch University, Perth, Australia

## Abstract

Chiropractic has endured a turbulent history, marked by tremendous advances in areas such as education and licensing while marred by interprofessional conflict and a poor public image. The prolonged interprofessional conflict was instrumental in shaping the culture of chiropractic. These obstacles have long-since been removed although there are lingering effects from them.

This article examines the chiropractic profession's history by dividing it into five Eras and suggests that there are three options available for the future of the profession. One: maintaining the status quo. Two: uniting under an evidence based scientific approach as partners in the health care delivery system that has buried the "one-cause, one-cure" sacred cow. The steps required to achieve this outcome are outlined. Three: openly dividing the profession into evidence based practitioners and subluxation based practitioners. Adopting this option would allow each branch of the profession to move forward in the health care delivery system unhindered by the other.

It is unclear which option the profession will choose and whether the profession is mature enough to follow option two remains to be seen. What is evident is that the time to act is now.

## Background

This article is based on the second FG Roberts Memorial Lecture, delivered by the author at the Annual Conference of the Chiropractic & Osteopathic College of Australasia, Melbourne, Australia 2011.

In the initial FG Roberts Memorial Lecture and the subsequent paper, Dr Reggars recounted some of the many accomplishments that the chiropractic profession has achieved over the past 30 years and how the changes have impacted on chiropractic and the chiropractic profession [1].

This paper reviews the origins of chiropractic from the unregulated 19^th ^century health care system in the United States of America and its frequently turbulent journey into the 21^st ^century. It is the author's contention that the culture of chiropractic as it stands is for a large part the result of external forces and that unless and until the profession recognizes how these forces have influenced its development, progression of chiropractic into the 21^st ^century health care system will not occur. Whilst some within the profession may consider this to be a good thing, it will be argued that the survival of the profession hinges upon its acceptance of science and seizing upon the tremendous opportunity ahead.

The author came into chiropractic with an exercise physiology background and graduated from Canadian Memorial Chiropractic College in 1982 with no understanding of 'The Big Idea' [2]. Following 24 years in private practice, a PhD in sociology and 7 more years in chiropractic academia, the author still wonders what all the fuss is about, but certainly recognizes the difficulties that The Big Idea has presented [3].

## Discussion

For the purpose of debate, the history of the profession will be divided into five distinct and at times overlapping eras:

1. The Era of Free trade in Medicine: 1860 ~ 1900

2. The Era of Prosecution: 1900 ~ 1950

3. The Era of Persecution: 1920 ~ 2000

4. The Era of Legitimation: 1960 - present

5. The Era of Opportunity: 2000 - present

### The Era of Free Trade in Medicine (1860 - 1900)

Chiropractic emerged out of what is recognized as The Era of Free Trade in Medicine (1860-1900). This was a time when all and sundry could ply their trade because there was no legislation governing health care in the United States of America [4,5].

Three predominant groups of "healers" were represented in the first half of the 19th century:

1. Orthodox medicine (physicians), whose principal treatment methods included bloodletting, blistering, purgatives (massive doses of compounds of mercury, antimony and other mineral poisons) and tonics (arsenical compounds);

2. Thomsonian/eclectics, whose principal treatment methods were botanical remedies, steam baths and rest; and

3. Homeopaths, whose principal treatment methods were homeopathic remedies, fresh air, sunshine, bed rest, proper diet, and personal hygiene [6].

Health care provision was not limited to members of these three groups however. This was also the time of the 'isms':

• Spiritualism

• Vitalism

• Mesmerism

• Mysticism

Add to the mix Christian Scientists, magnetic healers and manual therapists (osteopaths, chiropractors, bone setters) and we begin to appreciate the health care landscape of the time. It is noteworthy that, during this deregulated period, the United States became one of the healthiest nations in the world, boasting the world's lowest infant mortality rate [7]. This came about largely as a result of the work of a non-medical practitioner, Lemuel Shattuck. Shattuck's 1850 Report to the Sanitary Commission of Massachusetts has been hailed as one of the most significant documents in the history of public health and ultimately led to widespread adoption of public health and sanitation measures that were responsible for saving thousands of lives [8,9]. From within this pre-scientific era [10], chiropractic emerged in 1895 as an empirical "health science" interested in the spine and its possible relationship to disease states [11,12]. It is important to remember that chiropractic was not the only group interested in the notion of spinal irritation (aka: rachialgia) and that this was a very controversial subject [13,14].

The Era of Free Trade in Medicine gave way to the Golden Age of Medicine and The Golden Age of Scientific Advancement (1890 - 1950). Because chiropractic was heavily influenced by both of these Golden Ages, they will be considered briefly.

The Golden Age of Scientific Advancement largely drove the Golden Age of Medicine.

These ages brought with them what were considered miraculous medical breakthroughs, including vaccination and antibiotics. There were dramatic declines in the mortality rate for infectious diseases as well as increases in expected lifespan. No disease seemed incapable of being defeated by science. The medical profession enjoyed the benefits of these advancements with rapid progression to the top of the professional hierarchy and a concomitant increase in cultural authority [15,16].

This was also the era of medical education reform, which saw a science-based curriculum introduced concomitant with university affiliation as directed by the Flexner Report [17]. The American Medical Association's Medical Education Committee promoted the Flexner Report to the 168 institutions providing 'medical' education. Schools that agreed to adopt the Flexner recommendations received generous funding via the Rockefeller Foundation while schools that did not assent, simply ceased operation due to lack of funding. The result was that, by 1930, only 76 of the 168 medical schools remained [15]. In this manner, virtually all healing arts other than allopathic medicine were effectively eliminated, and the number of new medical graduates entering the profession was dramatically reduced from over 5440 per year to 3536 [15].

In part because of the rejection of science by a significant element within the chiropractic profession [18], and in part because the Flexner Report dismissed the chiropractors as "unconscionable quacks who should be dealt with by the public prosecutor and the grand jury" [17], the chiropractic profession bypassed the era of educational reform.

With Flexner's simple phrase, the chiropractic profession found itself mired in The Era of Prosecution, which was closely overlapped by The Era of Persecution.

### The Era of Prosecution: 1900 ~ 1950

Practically from its very origins in 1895, the chiropractic profession was in conflict with allopathic medicine [19]. The relationship between political medicine and chiropractic was described by Scotton as a "holy war between the forces of good and evil"[20] and Carter [21] referred to "the libel and slander campaign of dirty tricks" when describing political medicine's dealings with the chiropractic profession.

The Era of Prosecution began in 1902, less than a decade after Daniel David Palmer (DD) 'discovered' chiropractic. DD was charged with practising medicine without a licence in 1902 and 1903, however the charges were either dismissed, or the prosecution simply did not proceed [22]. In 1906, DD was the first chiropractor jailed following his conviction for practising medicine without a licence. He was fined $350.00 plus court costs, and incarcerated until the fine was paid. After serving 23 days, DD paid the fine and was released [23].

In the first thirty years of the chiropractic profession's existence, there were more than 15,000 prosecutions, about 20 percent of which resulted in incarceration [24]. In fact, "Go to jail for chiropractic" was the advice given to members of the Federated Chiropractors of California, and the Alameda County (California) Chiropractors' Association deemed it mandatory for its members to go to jail rather than pay a fine if convicted for practising medicine without a license [12]. On one occasion there was a mass arrest of 100 chiropractors in New York City [25]. In 1921 alone, 450 of the 600 California chiropractors chose jail over a fine following convictions for practising medicine without a licence [26] and such prosecutions continued well past mid-century [27].

With DD's conviction, his son, BJ Palmer, determined that a means of defending the profession was required and the Universal Chiropractor's Association (UCA) was founded, primarily as a protective association for all chiropractors. Shortly after its formation, the UCA officially condemned the "mixer" policies of the American Chiropractic Association, a rival association that had been formed by Langworthy in 1922 [28]. The condemnation was significant because, in a strictly pragmatic legal move, the UCA's legal counsel used Langworthy's textbook, *Modernized Chiropractic*, and the paradigm espoused therein, as the basis for successfully defending chiropractors charged with practising medicine without a licence [28].

The UCA's first case was the defence of Shegataro Morikubo, a graduate of the Palmer School and Infirmary of Chiropractic, who was charged in 1907 with the unlicensed practice of medicine, surgery and osteopathy. Upon learning of Morikubo's indictment, BJ Palmer travelled to Lacross, Wisconsin where the case was to be heard, and hired State Senator Tom Morris, LL.B., senior partner in the law firm of Morris and Hartwell, for the defence of Morikubo. The first step in the defence was to suggest to the prosecution that, because Morikubo had neither used any surgical procedure nor prescribed or used any drugs, the trial should be limited to the unlicensed practice of osteopathy. The prosecutor agreed and the trial proceeded on the sole charge of unlicensed practice of osteopathy. In presenting the defence arguments, Morris used *Modernized Chiropractic*, which stated that chiropractic had a separate and distinct philosophy and practice that was unlike any other method of treatment. He reinforced this argument by calling several witnesses, including practitioners trained in both osteopathy and chiropractic. Following a 30-minute deliberation, Morikubo was unanimously acquitted [29].

Using similar tactics, Morris oversaw the defence of more than 3300 chiropractors, with a win rate approaching 90 percent [28]. The Morris defense strategy was as simple as it was effective. It involved four clear concepts:

1. Chiropractic is not medicine; chiropractic has a "separate and distinct philosophy and practice".

2. Chiropractors do not diagnose, but analyze the spine for the cause of dis-ease.

3. Chiropractors do not "treat," but adjust the spine for the cause of dis-ease.

4. The Chiropractic profession has been built upon success in cases where medical doctors failed [28].

Morris has been acknowledged as the architect of "chiropractic philosophy" because of his demonstration of how to use chiropractic theories or philosophy in the courtroom to defend against charges of the unlicensed practice of medicine. In recognition of this, he was awarded the first "Philosopher of Chiropractic" (PhC) degree from BJ Palmer's chiropractic school in 1907 [30]. Following Morris' success in the Morikubo case, BJ Palmer embraced "chiropractic philosophy" with evangelical zeal, and thus the courtroom experience of Morikubo became a powerful determinant of the chiropractic profession's development [29].

It is noteworthy that the above interpretation has been questioned. Peters and Chance contend that Morikubo himself established the defense used by Morris [31]. It is clear that Morikubo and Morris consulted prior to the trial, and considering the recognition afforded Morris, it seems likely that Morris was the originator of the defense strategy used, and most certainly the record shows that he used it repeatedly and with great success.

Wardwell considered the Morikubo case the "third major event in chiropractic history", again because this was the point at which BJ Palmer began his "straight philosophical journey"[32]. During this journey, the chiropractic community developed and embraced a distinct lexicon and rationale toward health and its maintenance in order to emphasise the difference between medicine and chiropractic [33]. Thus the "philosophy of chiropractic" became "an unyielding dogma" [30].

### The Era of Persecution: 1920 - 2000

This Era began shortly after the Era of Prosecution and can be divided into an informal and a formal era.

Dr Morris Fishbein was labelled the "Medical Mussolini" and described as the most important non-chiropractor to influence the chiropractic profession because he was responsible for beginning the informal medical campaign against chiropractors and orchestrated the American Medical Association's (AMA) anti-chiropractic crusade for over fifty years [34]. From his position of power as editor of the Journal of the American Medical Association (JAMA) and secretary of the AMA (1924 to 1949) he determined the tone of articles appearing in JAMA and ensured that anti-chiropractic pieces regularly appeared [34].

In spite of, or perhaps because of having achieved market dominance, the AMA launched an "all-out war on chiropractic, the most successful of contemporary alternatives" [35]. This war constitutes the formal Era of Persecution. It began in 1962 with the adoption of The Iowa Plan and the formation of the Committee on Quackery to implement same [36]. Simply put, the Iowa Plan's overall goal was to "contain and eventually eliminate the cult of chiropractic as a health hazard in the United States" [36].

Chiropractors and chiropractic training institutions were in abundance during this time. To put things into perspective, by 1910 there were some 392 schools in the USA offering chiropractic training [37] with up to 16,000 practising chiropractors in the country [19]. This is in contrast to the 76 medical schools at the time.

The Committee on Quackery was well funded, and operated a highly successful campaign that was centred on three main strategies:

1. An ethics based boycott, which deemed it unethical for AMA members to have professional dealings with chiropractors;

2. Convincing other organizations to adopt or adapt the AMA's anti-chiropractic policy; and

3. Instituting a comprehensive political campaign to thwart chiropractic progress on several fronts, including but not limited to education, research and insurance funding [36].

The chiropractic profession's response to the formal campaign was launched in 1976 when five chiropractors, Drs Chester A Wilk, James W Bryden, Patricia B Arthur, Michael D Pedigo, and Steven Lumsden filed an antitrust lawsuit against the American Medical Association (AMA) and fourteen other defendants [38].

The Era of Persecution officially ended in the United States in 1987 when the presiding Judge, Susan Getzendanner, issued a permanent injunction order against the AMA because she correctly recognized that there would be lingering effects of the decades long AMA boycott: the culture of political medicine had become anti-chiropractic and the AMA would possibly re-offend [39,40].

The Iowa Plan was not confined to the United States. In Australia, the Australian Medical Association had adopted the Iowa Plan's ethics based boycott as well as a very negative 'exclusive dogma' policy on chiropractic. The Association was very active and successful in blocking the advancement of chiropractic within Australia [3]. While there was no trial or injunction order issued within Australia, the Australian Medical Association formally ended its ethics based boycott and rescinded its exclusive dogma policy on chiropractic following the intervention of the Australian Consumer and Competition Commission [41,42].

Clearly, opposition to the growing profession of chiropractic was formidable, prolonged and widespread. The profession's survival resembles the history of early social protest and reform movements in western history. Like the abolitionists, early chiropractors were victims of systematic persecution and often banished from one town to another. Similar to the feminists and suffragettes, pioneer chiropractors were often ostracised in the community [43]. While the persecution and prosecution might have been seen as impediments to the profession, the contrary was the case. Gibbons reminds us that "phase of real persecution and enforced poverty" afforded chiropractors "much of their identity and motivation" [44].

Importantly, from a sociological perspective, the sustained campaign by the AMA crystalized chiropractic culture into one with a unique lexicon and a siege mentality.

With the end of the Eras of Prosecution and Persecution came the Era of Legitimation during which the chiropractic profession enjoyed advancements on many fronts [32].

### The Era of Legitimation: 1960 - present

The Era of Legitimation saw inquiries into chiropractic - lots of inquiries - 18 in all. The New Zealand Royal Commission, the Webb Royal Commission in Australia, the LaCroix Royal Commission in Canada, the Layton Committee Report again in Australia to name a few.

The inquiries reached similar conclusions:

1) Chiropractors are well positioned to manage musculoskeletal disorders,

2) Claims by chiropractors to be able to positively affect visceral disorders are unsupported by evidence and constitute a major obstacle to integration of chiropractic into the health care system, and

3) Chiropractic education needs to improve.

The Era of Legitimation saw chiropractic education move into government funded universities; a proliferation of chiropractic research; legislation governing the practice of chiropractic in 90 jurisdictions throughout the world and unprecedented inter-professional cooperation [45].

There was an expectation that with registration and university based education, and the removal of other obstacles, the profession would unite, embrace science and take its *legitimate *place within the health care system and that the profession's cultural authority would be enhanced.

The reality is quite different. Instead, the siege mentality of the chiropractic profession largely remains and is compounded by memory deficit disorder, whereby it seems that these important historical facts have been lost, resulting in a significant portion of the chiropractic profession steadfastly continuing to espouse the original chiropractic doctrine [46] and make "unsubstantiated claims" [47,48].

The profession's public image remains in tatters with chiropractors consistently rating towards the bottom of ethics and honesty scales [49]. The 2006 Australian Reader's Digest most trusted professions poll placed chiropractors in 13^th ^place out of 35 professions [50]. While this is not a good result, in 2011 chiropractors do not even appear amongst the 45 professions listed, which placed real estate agents, car salesmen, politicians and tele-marketers in the last four spots [51]. While it is recognized that the Readers Digest poll is not the most scientific, this result is surely not a good sign for the chiropractic profession's cultural authority.

In the 2010 FG Roberts Memorial Lecture, Dr Reggars stated that the chiropractic profession is poised at the crossroads of marginalization and mainstream [1].

I say that the profession is at the crossroads of being relegated to the back blocks of health care provision (marginalization) or being recognized as the health care provider of choice for what bothers most of the people, most of the time.

#### So what is the problem?

Carl Sagan tells of a fire-breathing dragon living in his garage. When we look inside and cannot see his dragon he advises that it is invisible, and it cannot be detected with infrared because its fire is heatless; it leaves no footprints because it floats in the air and its body cannot be shown because it is incorporeal. Indeed, it cannot be detected by any known means. Sagan is the only one who knows it is there. Sagan asks what the difference is between a dragon that cannot be detected by any means and no dragon at all? If there is no way to disprove his dragon's existence, what does it mean to say that his dragon exists? The inability to invalidate his hypothesis is not at all the same thing as proving it true. Sagan advises that untestable claims and assertions incapable of disproof are worthless, even though they may excite us and fill us with wonder. To believe in Sagan's dragon in the absence of evidence is to do so on his authority alone [52].

The Association of Chiropractic Colleges defines the purpose, principles and practice of chiropractic as the finding and reduction of vertebral subluxations that "compromise neural integrity and may influence organ system function and general health". [53]. An internet search will advise that chiropractors have known about the dangers of subluxations for over one hundred years; that chiropractors are the only health professionals trained in the detection, and correction of the vertebral subluxation [54-56].

The problem is that there is no credible evidence that subluxations are any more real or dangerous [57,58] than Sagan's dragon. While Sagan may be deluding himself, he is unlikely to harm others because of his belief.

Chiropractic's problem is that subluxation based chiropractors [59] are not only deluding themselves, they are indoctrinating patients into believing in a purportedly dangerous mythical entity, and that without regular adjustments, patients will not only fail to reach their full potential, they will likely suffer serious health problems.

Some authors have suggested that this may be a threat to public health [60-62]. And this, at a time when the profession has just entered The Era of Chiropractic Opportunity.

### The Era of Chiropractic Opportunity: 2000 - present

Consider the following facts regarding musculoskeletal disorders (MSD). MSD are the leading cause of pain and disability in Australia. They are the second most common cause for a visit to a general medical practitioner and the third leading cause of expenditure in the Australian health care system: $3 billion per year ($8.2 million per day). Annually, MSD are responsible for 300000 hospital admissions, 15 million general medical practitioner office visits, and 13 million prescriptions, and MSD from motor vehicle accidents account for 25% of all health care expenditure [63,64].

This is just the beginning - these figures are only going to get worse. There is an aging population that requires more care for MSD, with a shortage of health care providers who are poorly distributed [65]. The solution to this problem has been known by health economists for decades: provider substitution [66]. Until recently, governments have not had the political will to implement this major policy reform. The health care landscape has changed sufficiently so that provider substitution is not only being considered a possibility, it has been implemented, examined and found to be a viable option that results in high levels of patient satisfaction and good clinical outcomes [67,68]. In the area of MSD, in particular low back pain, chiropractic care has long been shown to be an effective and cost effective alternative to conventional medical care [69,70].

Given these circumstances, it would appear that chiropractic is favorably positioned to play a strong role in the health care delivery system, but as Dr Reggars pointed out, this seems unlikely to happen given the current chiropractic landscape [1].

### The Way Forward

It seems that there are three options available to the profession:

1) Maintain the status quo.

2) Move forward as a united profession.

3) Divide the profession and let each faction fend for itself.

These options will be explored.

Option 1) While maintaining the status quo is an option, this course of action effectively prevents the profession from taking full advantage of the Era of Opportunity.

Option 2) In an ideal world, the profession would move forward in a united fashion and take its rightful place within the health care delivery system. To do this would require significant maturation on the part of the profession as demonstrated by taking the following steps:

1) Slay the one-cause (subluxation), one-cure (adjustment) sacred cow. While this may have been the original *a priori *Palmerian philosophy, in synchrony with similar concepts of the late-nineteenth century [71,72], it is not acceptable as a basis for patient care in light of today's knowledge-base [57]. Rather than bury the corpse, it should be taught as chiropractic history, warts and all. It would be a travesty to forget the profession's checkered past.

2) The profession needs to get out of the dogma-house and cease espousing Palmerian doctrine as gospel. While it was beyond distasteful to be labeled an unscientific cult that follows an exclusive dogma [3], when one considers the definitions of dogma^i ^and cult^ii^, the label may have been appropriate for part of the profession, and indeed may still be appropriate.

Escaping from the dogma house will require extraordinary cooperation amongst all aspects of the profession. Organizations such as the World Federation of Chiropractic and all major chiropractic associations will need to agree upon and adopt a position statement identifying the chiropractic subluxation as an historical construct that remains a hypothesis, which cannot form the basis for patient care until and unless there is a body of scientific evidence to support it. This is not dissimilar to what has occurred in the United Kingdom [73].

The position statement will need to be backed up by these organizations incorporating and enforcing appropriate statements into their ethical codes. Further, educational accrediting bodies will have to modify their standards to clearly state that subluxation is not a concept upon which to base patient care. From this, it follows that chiropractic teaching institutions will be required to restructure their curricula accordingly.

These moves will need to be reinforced by the actions of registration boards to deal harshly with misleading and deceptive advertising on the part of subluxation based chiropractors and the unconscionable conduct that typically occurs therewith. Specifically, registration boards must prohibit the common practice whereby potential clients present to a chiropractor for a musculoskeletal complaint, only to be convinced that they are in fact suffering from subluxation related disorders and require prolonged chiropractic care.

Insurance providers will need to alter their rebate guidelines to only provide payment for evidence-based care and to strike from their registers subluxation-based chiropractors.

All of this must happen and the public must be informed along the way.

Last, but by no means least, it will require each and every chiropractor to be intolerant of the nonsense that is out there.

The contract that the profession has with society and its patients is such that, in exchange for a significant degree of autonomy over education, licensing and credentialing, members of the profession are expected to maintain high standards of competence and moral responsibility and to subordinate financial gain to the higher values of responsibility to clients and the public interest [74,75].

If the profession is to gain the trust of the consuming public it must, of necessity, become truly self-policing.

Only in this way will chiropractic generate the cultural authority required for recognition as a group worthy of the title "Profession".

No longer can we cast a blind eye. By our silence we are giving consent.

Chiropractic is not about hope in a jar and philosophy is not about faith or dogma. Philosophy is about seeking truth, questioning one's beliefs and altering those beliefs in accordance with the best available evidence.

George W Bush once said:

I know what I believe. I will continue to articulate what I believe and what I believe - I believe what I believe is right [76].

While this type of thinking may be acceptable for a United States President, faith and dogma must not be the driving forces of our profession.

According to Alvin Toffler,

The illiterate of the 21^st ^century will not be those who cannot read and write, but those who cannot learn, unlearn, and relearn [77].

The chiropractic profession can choose to be illiterate, but it will do so at its peril. It is realized that thinking is hard and that those who are unaccustomed to thinking may even find it unpleasant. On the other hand, thinkers have always found it rather fun and there are no confirmed reports of anyone dying or being seriously injured by thinking. Thinking may necessitate changing our minds - which may not be a bad thing. At the very least the profession owes it to its patients.

Option 3) If a unified profession is not achievable, the other path is to split the profession into clear divisions: evidence based chiropractors and subluxation based chiropractors with each division openly declaring its hand. Evidence based chiropractors will become active contributors to mainstream health care while subluxation based practitioners will "opt out" and take their chances.

## Conclusion

The chiropractic profession emerged from an unregulated, unscientific era in health care. During its 116-year history, chiropractors have endured decades of prosecution and persecution and developed a siege mentality for survival. Following a protracted legal battle in the United States and a shorter non-courtroom stoush in Australia, the discriminatory anti-chiropractic behavior on the part of political medicine largely ceased. Sadly, to the detriment of the profession as a whole, some parts of the chiropractic profession remain mired in a nineteenth century mind-set. This is particularly perplexing considering the progress that the profession has made and the opportunities it now has.

This paper has explored these issues and suggested a way for the profession to proceed as a united group. This author would not be willing to wager on a unified future, but does know this: the future of the chiropractic profession is well and truly in its own hands and the time to act is now.

## Competing interests

The authors declare that they have no competing interests.

## Endnotes

^i ^Dogma: An authoritative principle, belief, or statement of ideas or opinion, especially one considered to be absolutely true.

The American Heritage Dictionary of the English Language, 4^th ^Edition. Houghton Mifflin Company. 2009

^ii ^Cult: A system for the cure of disease based on dogma set forth by its promulgator. The Merriam-Webster Online Dictionary. http://www.merriam-webster.com 2010
